# Multimodal Virtual Reality Assessment of Medication Effects in Attention-Deficit/Hyperactivity Disorder and Its Distinction From Depression: Cross-Sectional Study

**DOI:** 10.2196/85351

**Published:** 2026-03-02

**Authors:** Laura Asché, Julian Pakos, Hannah Schrage, Johanna Schuder, Luisa Jung, Dario Sanchez, Benjamin Selaskowski, Annika Wiebe, Alexandra Philipsen, Niclas Braun

**Affiliations:** 1 Department of Psychiatry and Psychotherapy University Hospital Bonn University of Bonn Bonn, North Rhine-Westphalia Germany

**Keywords:** actigraphy, adult ADHD, depression, eye tracking, hyperactivity, impulsivity, inattention, medication, virtual reality, diagnosis

## Abstract

**Background:**

Over the past 2 decades, virtual reality–based neuropsychological tasks have gained traction as tools for objectively assessing symptoms of attention-deficit/hyperactivity disorder (ADHD), offering enhanced ecological validity by simulating naturalistic environments. To complement realistic settings with an ecologically valid task, we recently developed the virtual email sorting task (VEST), which immerses participants into an office environment where they sort emails while being exposed to distractors.

**Objective:**

This study examined for the first time the VEST’s sensitivity to medication effects and its specificity in differentiating ADHD from other psychiatric disorders that share overlapping cognitive symptoms, such as major depressive disorder (MDD).

**Methods:**

A total of 23 unmedicated individuals with ADHD, 23 medicated individuals with ADHD, and 16 unmedicated individuals with MDD completed the VEST. During alternating distractor phases (DP) and nondistractor phases (NDP), we recorded the participants’ task performance; head, torso, and leg actigraphy; eye movements; and brain activity using functional near-infrared spectroscopy (fNIRS), and subjective symptom ratings. Correlational analyses of main objective and subjective task-related parameters were computed. Data were analyzed using mixed-design ANOVAs.

**Results:**

Processing time variability increased over time in participants with MDD and unmedicated ADHD as indicated by a group × block interaction (*P*=.04; η^2^_p_=0.10), while a group × phase interaction (*P*=.009; η^2^_p_=0.15) revealed that medicated participants with ADHD showed an increase during DP compared to NDP. Moreover, both ADHD groups exhibited increased head movements during DP compared to NDP (trend group × phase interaction: *P*=.09; η^2^_p_=0.08), an effect not observed in the MDD group, and higher rotation during DP in unmedicated individuals with ADHD (*P*<.001; η^2^_p_=0.23). Also, scores in 3 out of 4 subjective symptom intensity ratings of inattention, impulsivity, and emotional dysregulation were higher in at least 1 of the ADHD groups compared to the MDD group. No significant group differences were found in actigraphy measures of the arm and torso, fNIRS brain activity, or eye-tracking data. Regarding correlational analyses, inattention was correlated to off-task gaze (*r*=0.28; *P*=.03), hyperactivity with mean processing time (*r*=0.33; *P*=.01) and head movement (*r*=0.35; *P*=.006), and impulsivity with error rate (*r*=0.35; *P*=.006), and various significant correlations between objective parameters were found.

**Conclusions:**

Our findings highlight the potential of the VEST to differentiate between ADHD and MDD, as well as to detect medication-related effects within ADHD. The results underscore the value of multimodal and ecological assessment approaches with distractors in the evaluation of attentional and behavioral symptoms. The VEST may offer a standardized way to investigate complex behavior in mental disorders in research settings and, potentially, in clinical practice. However, further studies with greater statistical power are needed to confirm these findings.

**Trial Registration:**

German Clinical Trials Register DRKS00031259; https://www.drks.de/search/de/trial/DRKS00031259/details

## Introduction

For attention-deficit/hyperactivity disorder (ADHD), a neurodevelopmental disorder with an estimated prevalence of 5% in children and 2.58% in adulthood [1,2], the search for assessment tools to objectively assess its core symptoms of inattention, impulsivity, and hyperactivity [3] has been ongoing. At present, to assess adult ADHD, clinical interviews and self-report questionnaires are primarily used [4,5]. While these methods provide the highest diagnostic accuracy to date, they are influenced by various confounding factors, such as varying introspective skills and recall bias [6,7]. To overcome these limitations, researchers have explored various measures such as neuropsychological tests [8] and biomarkers [9]. Although such measures have successfully distinguished individuals with ADHD from healthy controls (HC) at the group level, they still lack sufficient clinical precision for accurate diagnoses at the individual level [8,10,11]. Moreover, studies often focus only on one or two domains such as performance or brain activity [12,13], and therefore may fail to encompass the full complexity of the disorder. Hence, to further increase the accuracy of the objective assessment of symptoms of ADHD and to fully depict the disorder, the search for suitable tools has been ongoing.

In recent years, virtual reality (VR)–based assessments have gained increasing popularity in the assessment of mental disorders in general [14], and especially in ADHD [15]. As outlined in more detail in previous works [16], VR-based assessments offer several advantages over traditional neuropsychological tasks. First, VR setups provide immersive features, such as the presentation of complex 3D environments that participants can interact with in a highly standardized manner [17]. Second, VR environments, such as virtual classrooms, are considered to be more ecologically valid than the experimental setups typically used in conventional neuropsychological tests [17]. Third, to examine the ADHD symptom of increased distractibility [3], realistic auditory and/or visual distractors can be incorporated into VR scenarios, allowing researchers to study their impact on various outcome measures. Fourth, VR can be integrated with a variety of physiological measures, including brain imaging, eye tracking, and actigraphy, enabling a multimodal assessment covering both behavioral and physiological indicators of ADHD [18,19].

One of the first VR-based assessment paradigms making use of these advantages for adults with ADHD was the virtual seminar room (VSR) [16,20], which was developed by our research group and inspired by a similar paradigm for children with ADHD, the virtual classroom [21]. In both paradigms, participants have to perform a continuous performance task (CPT) projected to the front of the virtual environment on a whiteboard or chalkboard, while the surrounding level of distraction is systematically varied by phases of high distraction (distractor phases [DP]) and low distraction (nondistractor phases [NDP]). Using the CPT, a neuropsychologically validated task for measuring sustained attention and impulsivity, in which participants are required to respond to target stimuli (eg, letters) and suppress responses to nontarget stimuli [22], was integrated into an ecologically valid classroom setting [21].

The multimodal VSR allowed for the measurement of task performance, brain activity via electroencephalography and functional near-infrared spectroscopy (fNIRS), head actigraphy, eye movements, and subjective symptom intensity. Differences were found, for instance, in CPT performance and actigraphy between individuals with ADHD and HC [20]. Based on separate training and test sets generated across multiple VSR studies [20,23], participants could be classified as ADHD or HC with 81% accuracy [18]. However, while the VSR represents a successful first step toward a more ecologically valid VR-based assessment of adult ADHD, within this scenario, it continues to rely on the highly abstract CPT.

To address this, we developed the virtual office room [24]. In this paradigm, the virtual environment is displayed in an open space office room in which the participants sit at a desk in front of a virtual desktop and perform a virtual email sorting task (VEST). In contrast to the CPT applied in the VSR, the VEST follows a more function-led than construct-driven approach, as the task is designed to simulate everyday goal-directed behavior within a realistic work context to increase its generalizability to real-world behavior [17]. Hence, more realistic stimuli material and task design are used than in the CPT. Moreover, as in the VSR, participants are exposed to alternating DP and NDP. In a first study using the VEST [24], we found a higher PTV in the ADHD group than in the HC, and a higher increase in head movement from NDP to DP in ADHD.

Beyond the differentiation between adults with and without ADHD, VR-based neuropsychological tests may also assist in assessing the effects of pharmacological treatments or other therapeutic interventions. This could, for instance, help in determining whether a treatment should be continued or adjusted, and which specific symptoms are being positively or insufficiently impacted. Up to now, the effect of medication has been investigated in 3 studies in children/adolescents using VR-based neuropsychological tests. All studies applied a virtual classroom scenario and reported lower reaction time variability [25-27], lower omission errors [25,27], as well as lower reaction times, commission errors, and decreased head movements [25] in medicated participants compared to unmedicated participants. In adults, we found a trend effect between medicated and unmedicated participants on omission errors in the VSR [20]. In summary, while VR-based neuropsychological tests have shown promising initial results in detecting medication effects, further research is needed, especially in adult populations.

To evaluate the potential of the VEST for ADHD in adulthood, it is crucial to also examine its capacity to differentiate individuals with ADHD from individuals with other psychiatric disorders that can involve similar cognitive impairments. As a first step in this approach, we decided to test a sample with major depressive disorder (MDD) in comparison to ADHD. In addition to the generally high comorbidity of the two disorders, they overlap in some cognitive domains [4,28], which is why it is of interest to see whether the multimodal procedure can be used to find parameter characteristics that are specific to ADHD. As of now, there have only been VR-unrelated studies investigating differences in neurocognitive function between individuals with ADHD and individuals with MDD. Potvin et al [29], for example, report similar cognitive performances of both groups in various measures of subjective and objective cognitive function, including working memory and processing speed, with lower performances than HC. Moreover, in a study by Paucke et al [30], individuals with MDD, with ADHD, and with both disorders, showed impairments in sustained attention compared to HC. In a study adopting the group design of Paucke et al [30] with a notably higher sample size, Van Hal et al [31] found impairments in attention and other executive functions in all clinical groups. With regard to actigraphy, while a plethora of studies have investigated movement in daily life in individuals with MDD [32], to the best of our knowledge, no studies have applied actigraphy measures during the performance of neuropsychological tasks. In general, MDD is associated with psychomotor disturbances such as psychomotor agitation and retardation [33], and individuals with MDD show lower activity than HC [32]. Considering differences in eye movement behavior in MDD compared to HC, studies have shown an increased maintenance of gaze on dysphoric and decreased maintenance of gaze on positive stimuli [34], in addition to slower reaction times in pro- and antisaccade tasks [35]. As of now, however, no studies have investigated the influence of distracting stimuli on task performance in MDD. When examining brain activity using fNIRS, the dorsolateral prefrontal cortex (dlPFC) appears to be of particular interest in ADHD, where reduced oxyhemoglobin (oxy-Hb) concentrations have been observed during inhibition tasks [36]. Similarly, studies in individuals with depression have also reported differences in activation levels in this brain region compared to HC during verbal fluency tasks [37]. Although both clinical groups show dlPFC hypoactivation across different cognitive tasks, it remains of interest to explore whether, particularly the component of distractor inhibition, reveals a pattern that is specific to ADHD compared to MDD and if so, how it is altered through medication.

The aim of this study was to investigate the VEST’s sensitivity to medication effects and its potential to differentiate individuals with ADHD from those with other psychiatric disorders. To this end, we compared 3 groups: unmedicated individuals with ADHD, medicated individuals with ADHD, and unmedicated individuals with MDD. Specifically, we examined performance on the VEST, eye movement behavior, brain activity measured via fNIRS, motor activity assessed through actigraphy (head, torso, nondominant arm, and opposite leg to nondominant arm), and self-reported symptom intensity. In addition to group comparisons, we investigated distractibility and time-on-task effects.

Based on the existing literature, we hypothesized that both ADHD groups would exhibit greater head and body movement than the MDD group.

Based on the heterogeneity and limited number of prior studies, we also formulated two research questions:

1. Can the VEST differentiate on any outcome parameter between medicated and unmedicated individuals with ADHD?
2. Can the VEST differentiate on any outcome parameter between unmedicated individuals with ADHD and unmedicated individuals with MDD?

## Methods

### Ethical Considerations

The study received approval from the ethics committee of the Faculty of Medicine at the University of Bonn (protocol number 105/23) and was preregistered on July 20, 2023, in the German Clinical Trials Register (DRKS00031259). All participants were informed in advance about the study’s objective and procedures, the use of technical equipment, and possible risks of participation such as motion sickness, and they provided written consent before taking part. Personal data such as names and addresses were recorded only on paper documents, including the consent form and the reimbursement form. A study ID was used for all data collection. Participants received a compensation of €40 (US $47.5) for full participation. Individuals who had to be excluded after the first session or who dropped out received prorated compensation.

### Participants

A total of 63 individuals participated in the study, including 24 medicated adults with ADHD, 23 currently unmedicated adults with ADHD, and 16 unmedicated adults with MDD. The participants were recruited in various ways: direct approach of suitable individuals at the ADHD outpatient clinic of the University Hospital Bonn, advertisements on the internal website, posting flyers in outpatient clinics, writing to self-help groups, and a press release followed by a radio report. Participants could take part in the study if they fulfilled either ADHD or MDD criteria but no other severe psychiatric disorder, such as psychosis or severe addiction disorder. Participants were not screened for comorbid autism spectrum disorder, as autism spectrum disorder did not constitute an exclusion criterion and its prevalence in the population with ADHD is markedly lower in adults compared to children and adolescents [38]. Furthermore, participants had to be aged between 18 and 50 years, have normal or corrected-to-normal vision, and no history of epilepsy or other severe neurological disorders, pregnancy, or skin irritation on the head.

### General Procedure

The study consisted of 2 separate appointments. During the first appointment, participants underwent a clinical assessment after providing informed consent. The assessment had 2 main objectives. First, to confirm the diagnoses of ADHD or MDD, depending on which group the participant should be assigned to, and to rule out the respective other condition. Second, to identify potential comorbidities that signified as an exclusion criterion. The assessment lasted approximately 1-2 hours and was conducted either in person at the psychiatry department of the University Hospital of Bonn or via a video call using the online platform Red Connect [39]. Two clinical interviews were administered: the Diagnostic Brief Interview for Mental Disorders (Mini-DIPS) [40] to screen for 17 mental disorders and the Integrated Diagnosis of ADHD in Adulthood [41] to confirm or rule out the diagnosis of ADHD. Both interviews are based on the criteria of the *DSM-5* (*Diagnostic and Statistical Manual of Mental Disorders* [Fifth Edition]) and *ICD-10* (*International Statistical Classification of Diseases, 10th Revision*) and were conducted in German. After the clinical interviews, participants completed various self-rating scales via the online survey tool SoSci Survey [42], including the Patient Health Questionnaire-9 (PHQ-9) [43], and ADHD Self-Rating Scale (ADHS-SB) [44]. Additionally, a self-constructed questionnaire was administered to collect various sociodemographic data and the medication status. If no exclusion criteria applied during the initial assessment, participants were invited to the second appointment. During this session, the actual VR experiment took place in the VR laboratory of the University Hospital of Bonn. Once prepared, 3 motion trackers were affixed to the participants. Next, they were seated, and preparations for fNIRS recording commenced. Prior to the start of data acquisition, participants were fitted with the head-mounted display (HMD), and the VR environment was launched. Within the VR, a brief eye-tracking calibration was conducted, followed by a 30-second acclimation period to allow participants to familiarize themselves with the virtual setting. After that, participants received task instructions and completed a short practice block, before the actual experiment started. This consisted of 2 blocks, each lasting 24 minutes. Symptom assessments were conducted following both blocks, and the Virtual Reality Sickness Questionnaire (VRSQ) [45] was administered at the conclusion of the session. Throughout the experiment, the software Lab Streaming Layer (Christian Kothe) [46] was used to record and synchronize all data streams, including task performance, fNIRS, actigraphy, and eye-tracking data. Following completion of the experiment, all measurement devices were removed, and participants were debriefed and dismissed. Since the same study supervisor conducted both the diagnostic assessment and the VR session, the study did not include experimenter blinding.

### Apparatus and Virtual Environment

**Figure 1:**
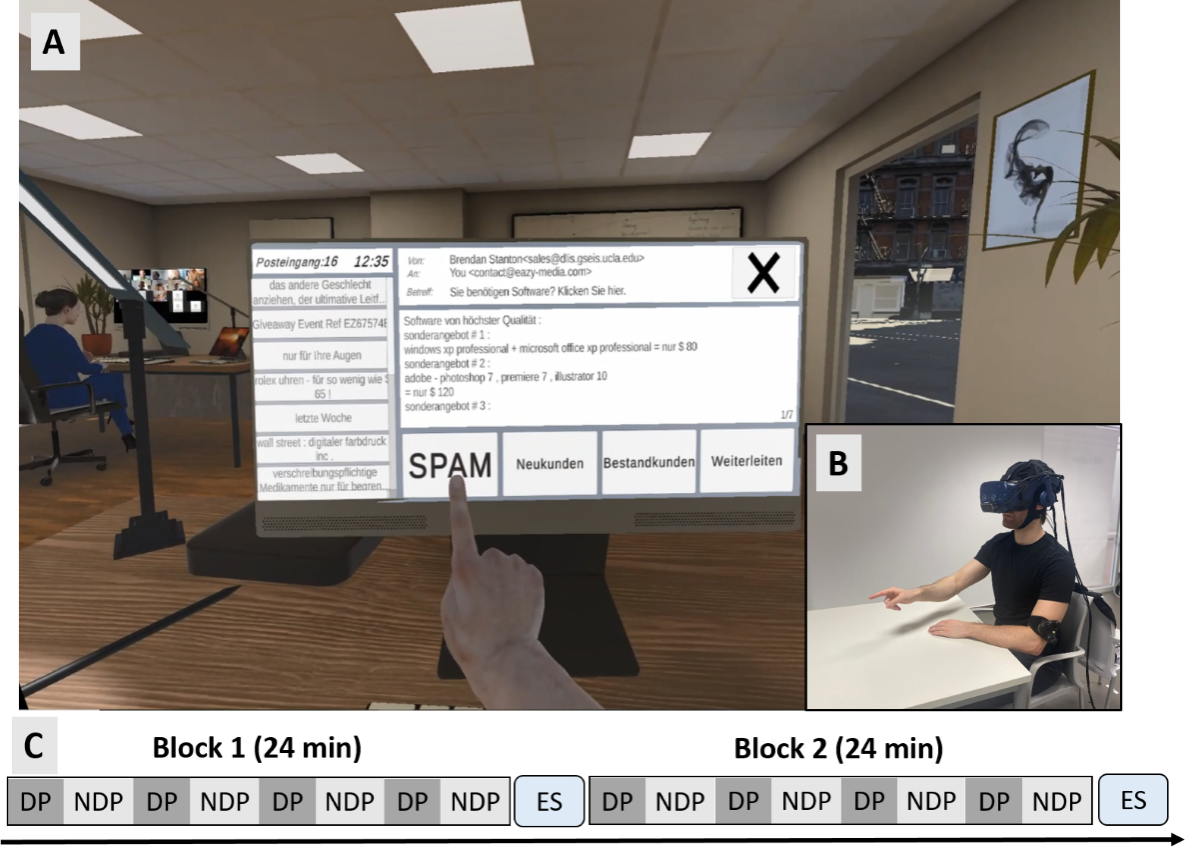
Technical setup, virtual environment, and experimental design. (A) First-person view of the virtual email sorting task in a virtual office. (B) Participant during the experimental session taking place in the virtual reality laboratory of the University Hospital Bonn. (C) Experimental timeline. DP: distractor phase; ES: experience sampling; NDP: nondistractor phase.

The VR scenario was presented via the HTC VIVE Pro Eye HMD (HTC Corporation). The VR scenario (Figure 1) was created using Unity 3D (version 2019.4.11f1; Unity Technologies) and coded in C# [24]. To enable controller-free interaction with the virtual environment, a Leap Motion Controller (Ultraleap) was used to track participants’ hand movements and online transfer them to a virtual 3D hand model (“Leap Motion Realistic Hands,” Unity Asset Store). The VR environment, entered via the HMD, depicted an open-plan office equipped with typical office supplies, furniture, and virtual colleagues (Figure 1; for further specifications of hardware and software, see Multimedia Appendix 1 [47,48]). In front of the participant, the VEST was displayed on a virtual computer screen.

### VEST

During the experiment, participants had to perform the VEST, a task that we developed and piloted in a previous study [24]). Compared to conventional CPTs, which often involve abstract stimuli, the VEST uses stimuli that mirror real-world office activities. In this task, participants assumed the role of a secretary in a marketing agency, where they were required to categorize incoming emails into 1/4 predefined categories: “spam,” “new customer,” “existing customer,” and “forward.” To ensure comparability with classical CPT paradigms, the task was structured such that 65% (389/599) of emails belonged to the spam category, making it the default response. Emails classified as “new customer” and “existing customer” each comprised 15% (90/599) of the total emails, while 5% (30/599) of emails fell into the “forward” category. Emails classified as “spam” were sourced from the Enron Spam Dataset (“Enron Spam Dataset,” 2015) but translated into German before usage. Emails for the remaining categories were composed by members of our research group and written from the perspective of a fictitious company or private individual and included both a subject line and a request relevant to a marketing agency. The emails contained clear identifying features corresponding to their respective categories, ensuring that each email had only one correct classification. In total, 599 mails were used in the VEST and presented in a randomized order.

The emails were displayed on a virtual computer screen in front of the participants (Figure 1). The screen simulated a touch interface, allowing participants to interact with it by using their virtual hands to select the appropriate category for each email (Figure 1). Emails did not expire after a certain time limit, so participants performed the task at their own pace—once an email was categorized, the next email would automatically open. Each block consisted of 4 DP and 4 NDP, each lasting 3 minutes and alternating. Consequently, over the course of the entire experiment, participants experienced 8 DP and 8 NDP. During each DP, 6 visual, auditory, or audiovisual distractors were presented in succession, each lasting 30 seconds. These distractors were randomly selected from a pool of 48 potential distractors. The order of the first phase in each block (ie, whether it was a DP or NDP) was counterbalanced across participants.

To assess the participants’ performance on the VEST, 3 distinct parameters were separately extracted for each DP and NDP: the average processing time and PTV (calculated by using the coefficient of variation) per email as measures of vigilance, and the error rate of misclassified emails as an indicator of inattention and impulsivity. For the statistical analysis, the mean values of each parameter were calculated across all DP and NDP per block.

### Experience Sampling

To evaluate participants’ subjective symptom experience, a VR-embedded user interface controlled via gesture input was presented immediately before block 1, between the 2 blocks, and at the end of block 2. For each block, participants were asked to rate their momentary levels of inattention, impulsivity, and hyperactivity [18,20,24], as well as their emotional dysregulation. The items were rated on a 7-point Likert scale, ranging from –3 to +3. For statistical analysis, only the subjective symptom reports collected after block 1 and block 2 were used. Following the final subjective symptom report, the VRSQ was presented on the same interface and Likert scale. The VRSQ includes 8 items assessing potential physical discomfort from VR exposure, from which a total score is calculated [45].

### Actigraphy Recording and Analyses

During task execution in the VR environment, the movements of the participant’s head, torso, arm, and leg were recorded. Head movement data were acquired via the HMD, with its position and rotation continuously tracked in the 3D space defined by the base stations. The movements and rotation of the torso, arm, and leg were captured using HTC Vive trackers (HTC Corporation), each of which was also tracked in the 3D space relative to its own dedicated base station. The placement of the trackers depended on the participants’ handedness, that is, for right-handed participants, 1 tracker was attached to the left upper arm, 1 around the torso, and 1 to the right ankle. For left-handed participants, the position of the ankle and upper arm were reversed. In line with our previous studies [16,20,24], all motion data were processed using a uniform analytical approach. First, the data were down sampled to ~10 Hz. Subsequently, the Euclidean distance between successive data points was calculated to quantify the change in position and rotation over time. The mean changes per task phase were then computed to summarize movement dynamics.

### Eye Tracking Recording and Analyses

To track the participant’s horizontal and vertical eye movements, the Tobii eye tracker (Tobii Technology) built into the HTC Vive Pro Eye was used (for further specifications of hardware and software, see Multimedia Appendix 1). Here, we tracked the participant’s duration of momentary gaze focus on the canvas on which the task was displayed on and the duration spent looking away from the canvas (off-task gaze) [24]. The momentary gaze focus time spent off-task was calculated for each NDP and DP.

### fNIRS Recording and Analyses

As in previous studies [16,20,24], the participants’ fNIRS brain activity was recorded during task performance by the NIRSport 2 system (NIRx Medical Technologies) and acquisition software Aurora fNIRS (NIRx Medical Technologies). We focused on oxy-HB in the dlPFC tracked by 2 channels (Fc1-Fc3, Fc2-Fc4; see also [20]), taking previous studies on ADHD using fNIRS [36] and spatial constraints into consideration (for further specifications of hardware and software used for recording and preprocessing, see Multimedia Appendix 1). For following statistical analyses, DP and NDP were first split into six 30-second units, equivalent to the length of distractor intervals in DP. Next, block averages across all units per phase were determined, and mean concentration of oxy-Hb was calculated for each phase. Lastly, each NDP was subtracted from its corresponding DP, and mean DP-NDP contrasts across all DP-NDP pairs were determined for each participant.

### Missing Data Handling

One participant was not able to conclude the experiment due to motion sickness in the second block and was excluded from analysis. For the VEST performance and head actigraphy, all remaining 62 datasets were complete and included; for eye tracking analysis, the sample was reduced to 61 datasets. Due to intermittent data transfer issues with the body trackers, some datasets were incomplete. If at least 3 out of 4 phases were successfully recorded, the phase average for each block was calculated using the 3 available values. However, datasets with more than 1 missing phase were excluded from the analysis. Using this approach, 58 datasets were included in the analysis of arm movements and 45 datasets in the analysis of torso movements. For leg movements, only 35 datasets remained, which led to the exclusion of this parameter from the analysis. Regarding the fNIRS data, 33 datasets were excluded for the left dlPFC and 39 for the right dlPFC due to insufficient data quality, leading to a reduced sample of n=29 and n=23, respectively. Since fNIRS was the only method used for analyzing brain activity in this study, the remaining datasets were still analyzed despite the decreased sample size. Multimedia Appendix 2 [49] presents descriptive summaries of missing data by modality and binary logistic regression analyses assessing whether missingness was associated with group, gender, and age.

### Statistical Analysis

For the majority of parameters of interest (eg, error rate; processing time; PTV; head, torso, and arm rotation and position; and off-task gaze), separate 3×2×2 mixed-design ANOVAs were conducted, with group as a between-subjects factor and block and phase as within-subject factors. For the experience sampling data, a 3×2 mixed-design ANOVA was performed with group and block as factors. Analyses of fNIRS and VRSQ data were conducted using 1-way ANOVAs with group as the factor. Furthermore, we conducted an exploratory correlational analysis of the objective parameters and subjective, task-related symptom intensity. For this purpose, we used aggregated values across phases and blocks. Because of the exploratory nature of the correlational analysis, we decided not to correct for multiple testing. Statistical significance was set at α<.05 and α between .05 and .10 was considered as a statistical trend level for all calculations.

Due to right-skewed distributions, several variables (eg, error rate, off-task gaze, and actigraphy measures for arm and torso) were subjected to square root transformations prior to analysis. Inattention scores exhibited left skewness; thus, these data were first mirrored and then square root transformed. Homogeneity of variances was assessed using Levene test, with *P*>.05 indicating acceptable homogeneity for all variables except inattention, for which homogeneity was not achieved despite transformation.

To control type I error inflation due to multiple testing, Bonferroni corrections were applied to the main effect of the 3-level group factor in the ANOVA, to interaction terms, and to all post hoc group comparisons. Effect sizes were interpreted according to Cohen [50] with partial eta squared (η^2^_p_) values of 0.01, 0.06, and 0.14 representing small, medium, and large effects, respectively for ANOVA results and Cohen *d* with values of 0.2, 0.5, and 0.8 representing small, medium, and large effects for post hoc comparisons.

## Results

All key ANOVA results are summarized in Multimedia Appendix 3.

### Sample

The sample consisted of 46 participants with ADHD, equally divided between the medicated and unmedicated groups, and 16 individuals with MDD. The 3 groups did not significantly differ in terms of gender, age, handedness, or level of education. Regarding self-reported clinical measures, the groups only differed on those that were specific to ADHD. Further demographic details can be found in Table 1.

**Table 1:** Demographic and clinical characterization of the 3 groups. *P* values indicate statistical group comparisons (1-way ANOVA or chi-square test).

Characteristics				Medicated ADHD^a^ (n=23)		Unmedicated ADHD (n=23)		MDD^b^ (n=16)		*P* value	
**Demographics**											
	Age (years), mean (SD; range)			29.65 (7.30; 22-44)		34.74 (10.59; 19-50)		31.75 (8.48; 20-50)		.16	
	**Gender, n (%)**										.07
		Men	9 (39.1)		9 (39.1)		2 (12.5)				
		Women	14 (60.9)		14 (60.9)		12 (75)				
		Nonbinary	N/A^c^		N/A		2 (12.5)				
	**Handedness, n**										.56
		Right	21		22		14				
		Left	1		1		2				
		Both	1		N/A		N/A				
	**Education, n**										.50
		Lower secondary education	4		6		6				
		Higher secondary education	11		12		5				
		University degree	8		8		5				
**IDA-R^d^ subscore, mean (SD)**											
	Inattention			18.43 (5.46)		20.96 (3.32)		8.56 (4.93)		<.001^e^	
	Impulsivity			6.78 (3.32)		8.00 (3.77)		2.44 (2.40)		<.001^e^	
	Hyperactivity			7.96 (3.20)		9.78 (4.13)		3.94 (2.89)		<.001^e^	
**ADHS-SB^f^ subscore, mean (SD)**											
	Inattention			14.74 (4.96)		17.35 (4.42)		6.81 (4.42)		<.001^e^	
	Impulsivity			5.22 (3.68)		6.48 (3.26)		1.94 (1.84)		<.001^g^	
	Hyperactivity			7.30 (3.27)		8.96 (4.61)		4.56 (2.94)		.003^e^	
**ADHD medication**											
	None, n (%)			N/A		23 (100)		16 (100)		N/A	
	Methylphenidate, n (%)			10 (43.48)		N/A		N/A		N/A	
	Lisdexamfetamine, n (%)			8 (34.78)		N/A		N/A		N/A	
	Nonstimulant, n (%)			3 (13.04)		N/A		N/A		N/A	
	Other, n (%)			2 (8.70)		N/A		N/A		N/A	
	PHQ-9^h^, mean (SD)			7.78 (5.57)		8.61 (4.95)		10.50 (7.40)		.37	
**BSL^i^, mean (SD)**											
	BSL-23			38.65 (15.92)		46.91 (14.91)		43.88 (21.13)		.26	
	BSL-VA^j^			59.96 (23.31)		60.13 (22.41)		56.07 (28.55)		.87	
	BSL-11			12.13 (1.82)		13.13 (2.65)		12.63 (1.96)		.31	
**WHO-QOL-BREF^k^, mean (SD)**											
	Physical health			68.63 (15.71)		69.72 (13.27)		69.20 (14.34)		.97	
	Psychological health			57.61 (17.21)		56.52 (15.83)		52.34 (21.51)		.65	
	Social relationships			64.13 (20.17)		60.87 (22.39)		63.02 (19.94)		.87	
	Environmental health			75.82 (14.14)		73.10 (13.18)		71.68 (12.58)		.61	

^a^ADHD: attention-deficit/hyperactivity disorder.

^b^MDD: major depressive disorder.

^c^N/A: not applicable.

^d^IDA-R: Integrated Diagnosis of ADHD in Adulthood.

^e^Post hoc comparisons revealed a significant difference between both ADHD groups and MDD, but not between the ADHD groups.

^f^ADHS-SB: ADHD Self-Rating Scale.

^g^Post hoc comparisons revealed a significant difference only between the unmedicated ADHD group and the MDD group.

^h^PHQ-9: Patient Health Questionnaire-9.

^i^BSL: Borderline Symptom List.

^j^BSL-VA: Borderline Symptom List-Visual Analogue Scale.

^k^WHO-QOL-BREF: WHO Quality of Life Short Version.

### VEST Performance

**Figure 2:**
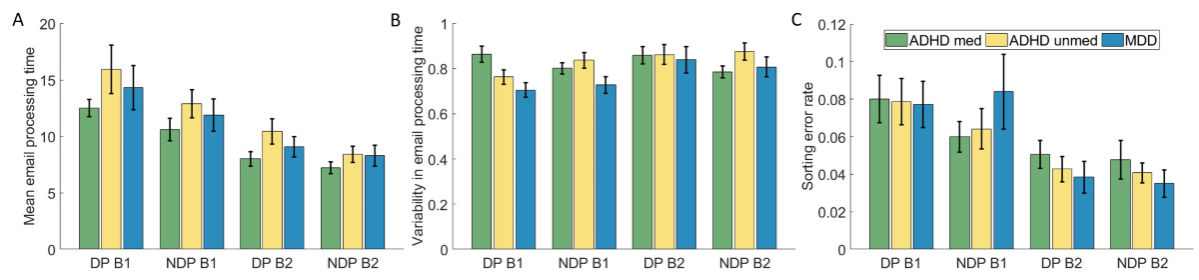
Results of the virtual email sorting task. (A) Mean processing time in seconds. (B) Processing time variability. (C) Error rate. Error bars indicate the standard error of the mean. ADHD: attention-deficit/hyperactivity disorder; B1: block 1; B2: block 2; DP: distractor phase; MDD: major depressive disorder; med: medicated; NDP: nondistractor phase; unmed: unmedicated.

Performance data of the VEST are presented in Figure 2. The analysis of mean processing time revealed significant main effects of block (*F*_1,59_=68.22; *P*<.001; η^2^_p_=0.54) and phase (*F*_1,59_=23.99; *P*<.001; η^2^_p_=0.29), but not of group (*F*_2,59_=1.49; *P*=.24). Mean processing time was higher in block 1 than block 2 (mean difference [MDiff]=4.48, 95% CI 3.40-5.57) and higher in DP than NDP (MDiff=1.86, 95% CI 1.10-2.61). In addition, there was a significant interaction of block × phase (*F*_1,59_=4.00; *P*=.05; η^2^_p_=0.06), which was not further interpreted.

For PTV, the analysis yielded a significant main effect of block (*F*_1,59_=8.39; *P*=.005; η^2^_p_=0.12), indicating a higher PTV in block 2 than block 1 (MDiff=–0.05, 95% CI –0.09 to –0.02). There were no significant main effects of phase (*F*_1,59_=0.46; *P*=.50) and group (*F*_2,59_=1.18; *P*=.32). However, the analysis revealed significant interactions of block × group (*F*_2,59_=3.35; *P*=.04; η^2^_p_=0.10), phase × group (*F*_2,59_=5.09; *P*=.009; η^2^_p_=0.15) and a trend for block × phase (F_1,59_=3.79; *P*=.06; η^2^_p_=0.06), which was not further interpreted. In the pairwise comparisons, we found a significantly higher PTV in block 2 than 1 in the unmedicated ADHD group (*P*=.03; *d*=0.39; MDiff=–0.07, 95% CI –0.13 to –0.01) and in the MDD group (*P*=.005; *d*=0.95; MDiff=–0.11, 95% CI –0.18 to –0.03), but not in the medicated ADHD population (*P*=.73). For phase × group, pairwise comparisons revealed a significant difference between DP and NDP in the medicated ADHD group (*P*=.02; *d*=0.78) with higher PTV in DP than NDP (MDiff=0.07, 95% CI 0.02-0.12), while for the unmedicated ADHD group (*P*=.09) and the MDD group (*P*=.88) there was no significant difference.

The analysis of square root–transformed error rates yielded a significant main effect of phase (*F*_1,59_=41.12; *P*<.001; η^2^_p_=0.41), indicating a higher error rate in DP than NDP (MDiff=0.02, 95% CI 0.01-0.02). Apart from that, no significant main effects of block (*F*_1,59_=1.79; *P*=.19) or group (*F*_2,59_=0.04; *P*=.96) or significant interactions were found.

### Actigraphy Results

#### Head

**Figure 3:**
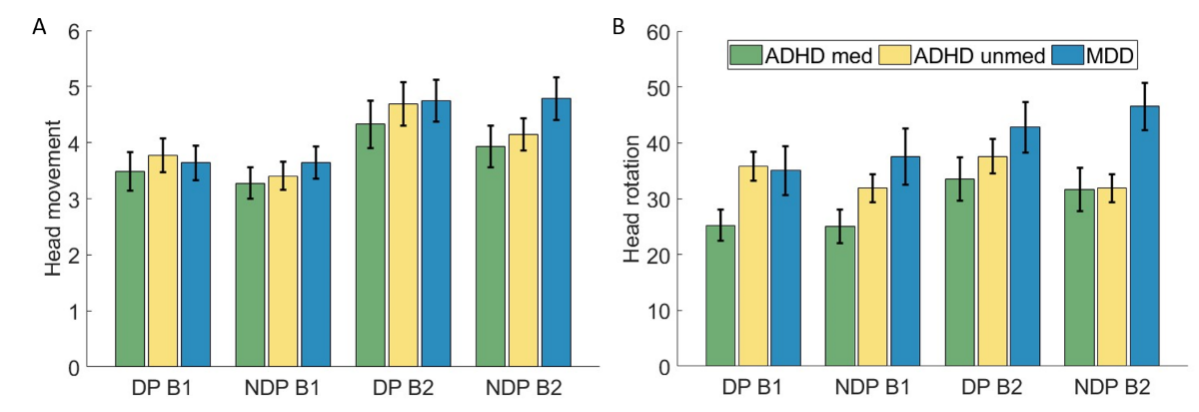
Results of head actigraphy analysis. (A) Head movement. (B) Head rotation. Error bars indicate the standard error of the mean. ADHD: attention-deficit/hyperactivity disorder; B1: block 1; B2: block 2; DP: distractor phase; MDD: major depressive disorder; med: medicated; NDP: nondistractor phase; unmed: unmedicated.

Results of head actigraphy analyses are depicted in Figure 3. The ANOVA for head movement revealed significant effects of block (*F*_1,59_=60.12; *P*<.001; η^2^_p_=0.51) and phase (*F*_1,59_=23.99; *P=.*005; η^2^_p_=0.13), but not of group (*F*_2,59_=0.48; *P*=.62). Movement was greater in block 2 compared to block 1 (MDiff=–0.90, 95% CI –1.13 to –0.67), and higher in the DP condition than in NDP (MDiff=0.25, 95% CI 0.08-0.42). A trend toward a phase × group interaction was also observed (*F*_2,59_=2.56; *P*=.09; η^2^_p_=0.08). Pairwise comparisons indicated significantly increased movement in DP compared to NDP for both ADHD groups (medicated: *P*=.03; *d*=0.42; MDiff=0.30, 95% CI 0.03-0.59; unmedicated: *P*=.001; *d*=0.66; MDiff=0.46, 95% CI 0.19-0.73), whereas no significant difference was found in the MDD group (*P*=.89).

For head rotation, a significant main effect of block emerged (*F*_1,59_=17.33; *P*<.001; η^2^_p_=0.23), with greater rotation in block 2 than block 1 (MDiff=–6.25, 95% CI –9.25 to –3.25). Also, there was a trend toward a group effect (*F*_2,59_=3.08; *P*=.05; η^2^_p_=0.10), though no significant effect of phase (*F*_1,59_=0.20; *P*=.66). Pairwise comparisons showed a trend for a difference between medicated individuals with ADHD and those with MDD (*P*=.05; *d*=0.72; MDiff=11.63, 95% CI –0.08 to 23.34). Additionally, a significant phase × group interaction was detected (*F*_2,59_=8.81; *P*<.001; η^2^_p_=0.23). Here, pairwise comparisons revealed significantly higher rotation in DP than NDP for the unmedicated ADHD group (*P*=.003; *d*=0.56; MDiff=2.83, 95% CI 1.00-4.65). By contrast, the reverse was observed for the MDD group (*P*=.006; *d*=0.65; MDiff=–3.10, 95% CI –5.29 to –0.91), and no significant difference was observed for the medicated ADHD group (*P*=.27).

#### Arms

Analysis of the square root-transformed arm movement and rotation data showed a significant block effect for both, arm movements (*F*_1,55_=38.93; *P*<.001; η^2^_p_=0.41) and arm rotations (*F*_1,55_=13.29; *P*<.001; η^2^_p_=0.20), with greater activity levels in block 2 than 1 (movement: MDiff=–0.30, 95% CI –0.40 to –0.21; rotation: MDiff=–0.30, 95% CI –0.46 to –0.13). No significant main effects were found for phase or group in either case (movement: phase with *F*_1,55_=0.02; *P*=.90, group with *F*_1,55_=0.53; *P*=.59; rotation: phase with *F*_1,55_=0.31; *P*=.58, group with *F*_1,55_=1.73; *P*=.19). For arm rotation, an additional interaction between block and phase was observed (*F*_1,55_=5.41; *P*=.02; η^2^_p_=0.09), but not further explored.

#### Torso

For square root–transformed torso movement, the ANOVA revealed a significant main effect of block (*F*_1,42_=14.73; *P*<.001; η^2^_p_=0.26) indicating increased movement in block 2 compared to block 1 (MDiff=–0.15, 95% CI –0.24 to –0.07). No significant main effects of phase (*F*_1,42_=0.01; *P*=.91) or group (*F*_1,42_=0.32; *P*=.73) were observed.

For rotation, the ANOVA revealed a significant main effect of phase (*F*_1,42_=4.30; *P*=.04; η^2^_p_=0.09), with greater rotation observed during the DP than NDP (MDiff=0.10, 95% CI 0.00-2.00). No significant effects of block (*F*_1,42_=0.56; *P*=.46), group (*F*_1,42_=0.68; *P*=.51), or their interactions were found.

### Eye Tracking

**Figure 4:**
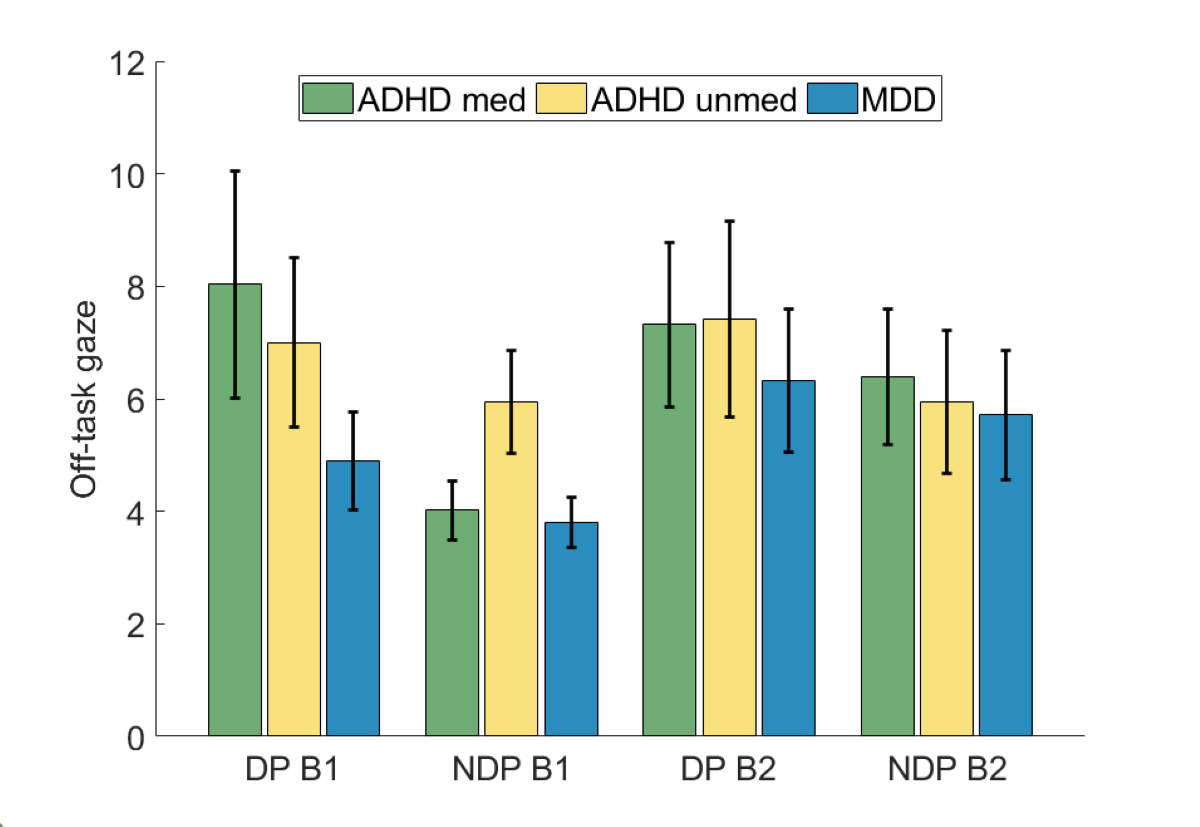
Results of the eye tracking analysis (off-task gaze time). Bar plot depicts logarithmically transformed data. Error bars indicate the standard error of the mean. ADHD: attention-deficit/hyperactivity disorder; B1: block 1; B2: block 2; DP: distractor phase; MDD: major depressive disorder; med: medicated; NDP: nondistractor phase; unmed: unmedicated.

Eye tracking data are depicted in Figure 4. The ANOVA for square root–transformed off task gaze data revealed a significant main effect of phase (*F*_1,58_=4.72; *P*=.03; η^2^_p_=0.075), in that across groups, there was a higher off task gaze in DP than NDP (MDiff=0.31, 95% CI 0.02-0.59). Apart from that, no main effect of block (*F*_1,58_=0.50; *P*=.48) or group (*F*_1,58_=1.16; *P*=.32) and no significant interactions were observed.

### fNIRS

One-way ANOVAs revealed no significant group differences in DP-NDP oxy-Hb contrast on either channel of interest with *F*_1,28_=0.29; *P*=.73; η^2^_p_=0.02 for the left dlPFC and *F*_1,22_=0.06; *P*=.94; η^2^_p_=0.07 for the right dlPFC.

### Experience Sampling

**Figure 5:**
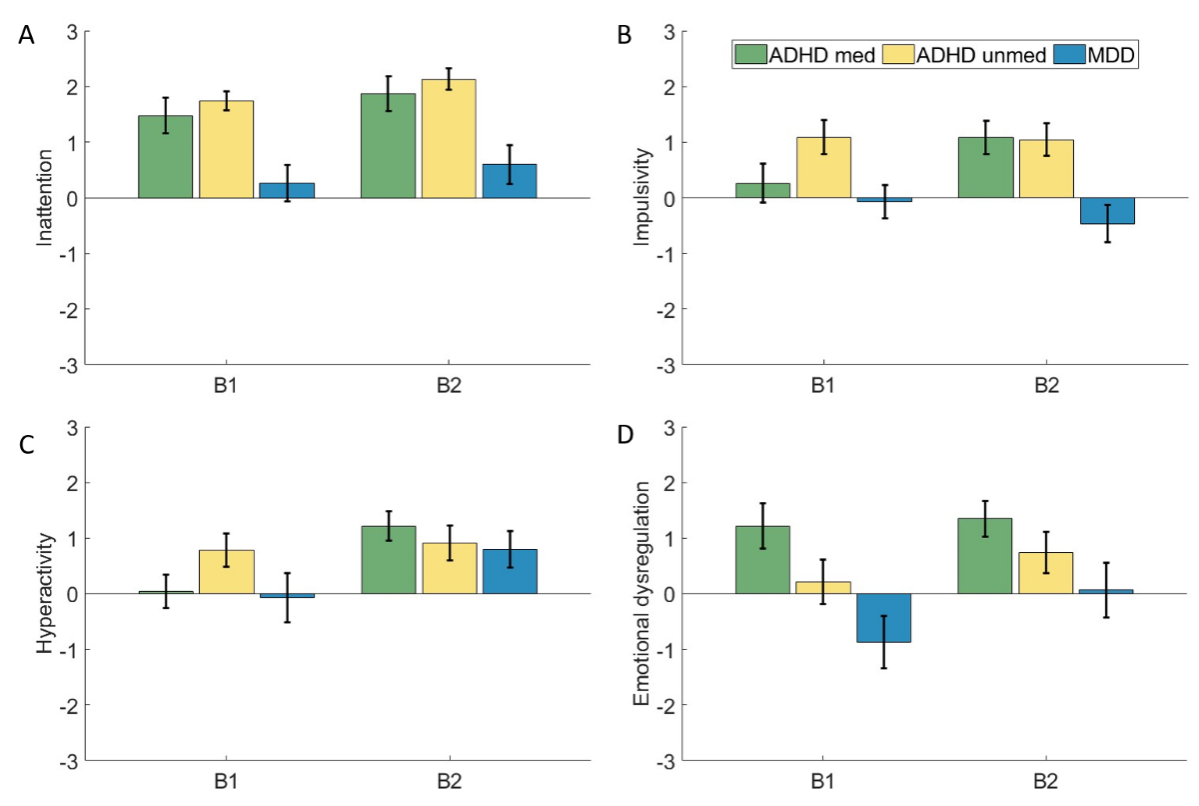
Results of experience sampling. (A) Inattention rating after block 1 and block 2. (B) Impulsivity rating after block 1 (B1) and block 2 (B2). (C) Hyperactivity rating after B1 and B2. (D) Emotional dysregulation rating after B1 and B2. Error bars indicate the standard error of the mean. ADHD: attention-deficit/hyperactivity disorder; MDD: major depressive disorder; med: medicated; unmed: unmedicated.

Results of the participants’ reported ADHD symptom intensity during and after task performance are depicted in Figure 5. The ANOVA of self-reported square root–transformed inattention intensity revealed a significant main effect of block (*F*_1,58_=9.52; *P*=.003; η^2^_p_=0.14), with higher scores in block 2 than 1 (MDiff=–0.09, 95% CI –0.03 to –0.15). A significant main effect of group also emerged (*F*_1,58_=9.28; *P*<.001; η^2^_p_=0.24). Pairwise comparisons showed significantly higher inattention scores in both medicated (*P*=.007; *d*=0.91; MDiff=0.27, 95% CI 0.48-0.06) and unmedicated (*P*<.001; *d*=1.68; MDiff=0.36, 95% CI 0.57-0.15) participants with ADHD compared to those with MDD, while the ADHD groups did not differ significantly (*P*=.69). No significant block × group interaction was found.

Regarding subjective impulsivity, a significant main effect of group was found (*F*_2,58_=6.16; *P*=.004; η^2^_p_=0.18). Post hoc comparisons indicated that unmedicated participants with ADHD reported significantly higher impulsivity than participants with MDD (*P*=.003; *d*=1.22; MDiff=1.40, 95% CI 0.42-2.38). Additionally, there was a trend (*P*=.10; *d*=0.73; MDiff=0.88, 95% CI –0.11 to 1.86) for higher impulsivity scores in medicated participants with ADHD than in participants with MDD, while the ADHD groups did not differ significantly (*P*=.44). No significant main effect of block was found (*F*_1,58_=0.38; *P*=.54), nor was there a significant interaction between group and block.

For self-reported hyperactivity, a significant main effect of block was observed (*F*_1,58_=16.87; *P*<.001; η^2^_p_=0.22), indicating higher hyperactivity levels in block 2 compared to block 1 (MDiff=–0.72, 95% CI –1.08 to –0.37). There was no significant main effect of group (*F*_2,58_=0.54; *P*=.59). However, a trend-level interaction between block and group emerged (*F*_2,58_=2.53; *P*=.09; η^2^_p_=0.08). Pairwise comparisons revealed significant increases in hyperactivity from block 1 to block 2 in the medicated ADHD group (*P*<.001; *d*=0.68; MDiff=–1.09, 95% CI –1.65 to –0.52) and MDD group (*P*=.02; *d*=0.70; MDiff=–0.87, 95% CI –1.56 to –0.17), whereas hyperactivity levels remained stable across blocks in the unmedicated ADHD group (*P*=.44).

For emotional dysregulation, ANOVA revealed a significant main effect of block (*F*_1,58_=9.20; *P*=.004; η^2^_p_=0.14), with higher scores observed after block 2 compared to block 1 (MDiff=–0.53, 95% CI –0.88 to –0.18). A significant main effect of group also emerged (*F*_1,58_=3.74; *P*=.03; η^2^_p_=0.11). Post hoc comparisons indicated that the medicated ADHD group reported significantly higher emotional dysregulation than the MDD group (*P*=.03; *d*=0.94; MDiff=1.55, 95% CI 0.15-2.96). No significant differences were found between the two ADHD groups (*P*=.86) or between the unmedicated ADHD and MDD groups (*P*=.24).

### VR Sickness

Across groups, mean VRSQ scores were in a low positive range (mean 0.31, SD 1.23), indicating a low level of motion sickness during the experiment. The conducted 1-way ANOVA revealed no significant differences between groups (*F*_2,60_=1.04; *P*=.36).

### Correlation Analyses Between Modalities

Core findings of the correlational analysis are presented in Table 2, whereas results of all computed correlations with respective sample sizes are available in Multimedia Appendix 4. Regarding associations between task-related symptom intensity and psychophysiological markers, inattention was correlated with off-task gaze (*r*=0.28; *P*=.03). Hyperactivity correlated with both mean error rate (*r*=0.34; *P*=.008) and mean processing time (*r*=0.33; *P*=.01), as well as with head movement (*r*=0.35; *P*=.006) and head rotation (*r*=0.30; *P*=.02). Analyses further showed a correlation between impulsivity and mean error rate (*r*=0.35; *P*=.006), and between emotional dysregulation and off-task gaze (*r*=0.29; *P*=.02). For the correlational analysis between the objective measures, we found, among other things, a significant correlation between off-task gaze and mean PTV (*r*=0.33; *P*=.01), head movement (*r*=0.43; *P*<*.*001), and arm movement (*r*=0.40; *P*=.002). Additionally, to its significant correlation to off-task gaze, mean PTV was also found to be correlated to head movement (*r*=0.35; *P*=.006) and rotation (*r*=0.26; *P*=.04). Furthermore, we also found a significant association between head rotation, mean processing time (*r*=0.30; *P*=.02), and head movement (*r*=0.51; *P*<.001).

**Table 2:** Correlation analysis among the study variables.

Variable			Mean PTV^a^		Mean error		Mean PT^b^		Off-task gaze		Head movement		Head rotation		Impulsivity		Hyperactivity		Inattention		Emotional dysregulation
**Mean PTV**																					
	*r*	—^c^																			
	*P* value	—																			
**Mean error**																					
	*r*	0.079		—																	
	*P* value	.54		—																	
**Mean PT**																					
	*r*	0.174		0.069		—															
	*P* value	.18		.60		—															
**Off-task gaze**																					
	*r*	0.325		0.251		0.056		—													
	*P* value	.01		.05		.67		—													
**Head movement**																					
	*r*	0.348		0.080		0.236		0.423		—											
	*P* value	.006		.54		.07		<.001		—											
**Head rotation**																					
	*r*	0.262		0.038		0.297		0.069		0.505		—									
	*P* value	.04		.77		.02		.60		<.001		—									
**Impulsivity**																					
	*r*	0.115		0.346		-0.066		0.181		–0.006		0.004		—							
	*P* value	.38		.006		.62		.16		.96		.97		—							
**Hyperactivity**																					
	*r*	0.202		0.339		0.325		0.071		0.345		0.298		0.224		—					
	*P* value	.12		.008		.01		.59		.006		.02		.08		—					
**Inattention**																					
	*r*	0.176		0.186		0.006		0.280		0.161		–0.079		0.500		0.377		—			
	*P* value	.18		.15		.97		.03		.21		.54		<.001		.003		—			
**Emotional dysregulation**																					
	*r*	–0.03		0.147		–0.215		0.294		0.047		–0.047		0.395		–0.061		0.308		—	
	*P* value	.82		.26		.10		.02		.72		.72		.002		.64		.02		—	

^a^PTV: processing time variability.

^b^PT: processing time.

^c^Not applicable.

## Discussion

### Principal Findings

In this study, we applied our virtual office room scenario with its VEST to a sample of medicated participants with ADHD, unmedicated participants with ADHD, and unmedicated participants with MDD. Using a multimodal approach, we explored the paradigm’s potential to detect differences in various outcomes across the 3 groups, extending previous research on VR-based assessment methods [20,24]. While we expected more movement in the ADHD groups than in the MDD group, only partial evidence supported this hypothesis. Unmedicated individuals with ADHD showed a distraction-induced increase in head rotation, whereas those with MDD showed a decrease in rotation during distraction. A trend-level effect also indicated more head movement during distraction in both ADHD groups compared to the MDD group. Additionally, regarding our research questions, we found an increase in PTV in unmedicated individuals with ADHD and in those with MDD, but not in medicated individuals with ADHD. Furthermore, we found an increase in PTV during distraction in medicated individuals with ADHD. At least one of the ADHD groups also reported higher task-related levels of inattention, hyperactivity, and emotional dysregulation than the MDD group. No differences between groups were found in eye movement, brain activity in the dlPFC as measured via fNIRS, and movement of the arms and torso. These findings indicate differences between the investigated groups in some of the parameters measured, and distinct responses to distractors and time-on-task, which will be examined in more detail in the following discussion.

Investigating task performance, we found a significant increase in PTV over time in participants with unmedicated ADHD and participants with unmedicated MDD, but not in medicated participants with ADHD. This finding indicates a time-on-task decline of sustained attentional performance or vigilance in unmedicated ADHD and MDD. This result aligns partially with previous research using VR-based CPTs in children, reporting lower reaction time variability in medicated compared to unmedicated individuals with ADHD [25-27]. Moreover, a meta-analysis by Kofler et al [51] found significant effects of stimulant treatment on reaction time variability, whereas no consistent reaction time variability differences were found between adults with ADHD and other clinical populations, including MDD. Our results indicate that medication helps maintain attentional focus over time in ADHD and that unmedicated individuals with ADHD or MDD show a comparable decline in attentional performance.

Additionally, medicated participants with ADHD showed a significantly higher PTV in DP than NDP. Although the effect was observed only in the medicated ADHD group, this finding suggests that the task is sensitive to distractor-specific influences on performance variability. The absence of the effect in the unmedicated ADHD group remains unclear. One possible explanation concerns the measure of variability; while the coefficient of variation is a common metric for reaction time variability, some argue that controlling for mean reaction time may be inappropriate, particularly in ADHD populations [52]. In this study, while not significantly different, unmedicated individuals with ADHD showed descriptively higher mean processing time than the medicated ADHD and the MDD group. Additionally, it is unclear to what extent processing time for emails is comparable to reaction times measured in tasks such as CPTs, which may also suggest the need for an alternative measure of variability.

Of note, no group differences emerged in mean processing time and error rates. This means that we were unable to use the VEST to map the comparatively slower information processing of individuals with MDD that has been observed in other neuropsychological tasks [53]. The error rate, which can be considered a measure of impulsivity, also showed no group differences. This may be due to the task itself, as the stimulus remains visible until a response is made rather than disappearing after a brief interval. It is also possible that, alongside the well-established impulsivity in ADHD, individuals with MDD exhibit impulsivity problems that reduce group differences, which has previously been shown [54]. However, it should be noted that the previous study likewise found no differences in error rates between ADHD and HC [24].

In addition, we observed a longer processing time in DP compared to NDP across groups. While this finding suggests that the experimental induction of distractibility was generally effective in observing reduced attentional performance across all groups, the distractors did not appear to elicit symptom-specific patterns of distractibility that would differentiate between diagnostic groups or between medicated and unmedicated participants.

We hypothesized that both ADHD groups would exhibit increased movement compared to the MDD group. This hypothesis is based on prior research that suggested that the overall activity level of individuals with MDD is decreased compared to those with ADHD. In our task, we measured specific body movements while sitting during task performance, which participants with ADHD and MDD did not differ in. Hence, differences might be found in overall motoric activity but not necessarily in physical activity during cognitive tasks. Nonetheless, the hypothesis was partially supported by a trend-level increase of movement during DP in both ADHD groups, but not in the MDD group. Similarly, we previously found a more pronounced increase in head movements from NDP to DP in ADHD compared to HC [24]. This pattern may reflect core symptomatology of ADHD, particularly hyperactivity and distractibility. If replicated, this differential movement response to environmental distractors may serve as a clinically relevant marker to distinguish between ADHD and MDD populations. In line with this, unmedicated participants with ADHD also displayed a significantly greater head rotation in DP than NDP. This finding further supports the potential use of actigraphy-derived motion parameters as objective, ecologically valid markers for differential diagnosis. By following up the phase and group interaction, we also found a higher head rotation in NDP compared to DP in participants with MDD. While seemingly counterintuitive at first, this finding might be due to the paradoxical effects of distractors. Prior research has presented evidence that distractors can sometimes facilitate performance [55,56], depending on the task characteristics and properties of the distractors, and the specific population explored, including MDD and ADHD [57,58]. Given the unclear directionality and inconsistency with baseline expectations, further research is needed to clarify the mechanisms behind this effect.

The increase in movement of the head, arms, and torso across groups is in line with our previously reported increase in head movement over time in the same paradigm in HC and individuals with ADHD [24]. Increased body movement during sustained attention tasks may reflect a compensatory mechanism to maintain attention [59,60] and can be viewed as an adaptation to the demands of prolonged sustained attention, such as decreasing arousal or accumulating fatigue.

As pertains eye tracking, we found a reduced focus on the task in phases with distractions compared to phases without distractions, but no group differences. Regarding ADHD medication effects, this null finding aligns with our previous study [20], which also revealed no significant differences in eye movement patterns between these 2 subgroups. We also did not find any differences between ADHD and MDD, indicating that all groups spent a similar amount of time looking at the task. Nonetheless, it remains possible that subtle effects exist but were not detected due to limited statistical power resulting from relatively small sample sizes.

In the fNIRS analysis, we did not observe any group differences in the DP-NDP contrast in the dlPFC. This may partly be due to the loss of data caused by technical issues, reducing the analyzable sample on both channels of interest to 29 and 23, respectively. In contrast to traditional inhibitory control tasks, where reduced activation in the dlPFC is consistently reported in individuals with ADHD compared to HC [36], we found no difference between medicated and unmedicated individuals with ADHD. It is also possible that the type of inhibitory control required in classical tasks is different from inhibiting distractors, or alternatively, that the distractors used in our study were not strong enough to elicit such effects [24].

Pertaining to self-reports of task-related symptom experience, both ADHD groups reported higher levels of task-related inattention and impulsivity compared to the MDD group. Assuming that our PTV measure partly captures inattention, this contrasts with our finding that unmedicated individuals with ADHD or MDD showed greater PTV increases than medicated individuals with ADHD. Hence, there appears to be a partial discrepancy between behavioral and self-reported measures, which could mean that they capture different aspects of attentional dysfunction or be attributed to a greater sensitivity of PTV as an index of inattention. For impulsivity, although elevated levels have also been reported in MDD [54], the higher scores observed in the unmedicated ADHD group and, at a trend level, in the medicated ADHD group are consistent with the classification of impulsivity as a core symptom of ADHD. In terms of emotional dysregulation, only the medicated ADHD group reported higher scores than the MDD group, whereas both ADHD groups showed comparable levels to each other. Difficulties in emotion regulation are frequently reported in association with ADHD [61], and in our sample, medication use does not appear to have exerted a beneficial effect, with descriptively higher scores in the medicated group. The MDD group showed the lowest scores, which differed significantly from those of the medicated ADHD group. Because participants in our MDD group were affected by mild to moderate depressive symptoms that did not currently require medication, it is conceivable that they were able to use emotion-regulation strategies such as acceptance. Nevertheless, previous research has shown that individuals with MDD, even when remitted, still tend to suppress emotions, which could also manifest in lower scores [62]. Importantly, emotional dysregulation is a construct that includes various dimensions such as emotional reactivity and intense experience of emotion, but also decreased emotional awareness [63]. Therefore, it is possible that the item used to assess task-related emotional dysregulation might have only encapsulated one facet of emotional dysregulation. Also, it should be noted that, irrespective of absolute levels, all 3 groups reported values in the positive range of emotional dysregulation after the second block.

No group differences emerged in self-reported hyperactivity, which contrasts with the distractor-induced differences detected via head actigraphy, but aligns with the null results from actigraphy-derived torso and nondominant arm movements as discussed above.

Lastly, we conducted an exploratory correlational analysis of the task-related symptom intensity and the objective parameters we measured. Two key points emerged: first, self-reported, task-related intensity of inattention, hyperactivity, impulsivity, and emotional dysregulation were each correlated with different objective measures. For instance, inattention and emotional dysregulation were associated with higher levels of off-task gaze; impulsivity was associated with a higher error rate; and hyperactivity was associated with increased head movement, higher error rate, and longer processing time. Although exploratory, this pattern might suggest that a multimodal assessment combining task performance, eye movements, and body movements (particularly head movement) may capture distinct symptom dimensions of ADHD. Second, off-task gaze and head movement and rotation showed a correlation with mean PTV, and, in the case of head rotation, with mean processing time. While these findings present a first glance into the potential relationships of different behavioral modalities during the VEST, the causal relationship of these parameters needs further investigation.

### Limitations and Future Directions

While the VEST was specifically designed to increase ecological validity compared to traditional neuropsychological tasks by applying a function-led design approach, it would be of interest to investigate the specific cognitive domains it draws upon [24]. Therefore, future studies should examine its associations with established (neuropsychological) measures, for example, attention and reading comprehension. Additionally, although the VEST is designed to simulate a realistic setting and task, a systematic investigation of its ecological validity, meaning the correspondence between behaviors exhibited in the virtual environment and in real-world settings [64], is still needed. While it would be very challenging to assess eye movement behavior, brain activity, and performance measures during the participants’ work to evaluate the equivalency of behavior in the virtual office room to real-world behavior, several alternative approaches could be applied. For example, cognitive functioning at work could be assessed using questionnaires [65,66]. Moreover, office-based participants could be asked to wear a mobile eye tracker and an actigraph during work sessions, while ambient noise levels would be simultaneously measured to assess auditory distractions. This would allow investigation of the relationship between distractor-related shifts of attention and movements in the virtual office room and real life.

Further, although the total sample comprised 62 participants, the sizes of the diagnostic subgroups, particularly the MDD group, were limited. As indicated by a sensitivity analysis, our sample consisting of 63 participants provided 80% power to detect large main effects (η^2^_p_=0.14, as calculated via G*Power, Heinrich Heine University Düsseldorf [67], based on the harmonic mean group size). Consequently, small- to medium-sized differences between the investigated population may not have been detected. Future studies with larger and more balanced samples are necessary to replicate and extend these results. For example, to reliably detect medium main effects, at least 50 participants per group would be needed. Furthermore, while the MDD diagnosis of participants in the MDD group was confirmed using the structured clinical interview Mini-DIPS, some MDD participants only reported mild depressive symptoms in the PHQ-9 [43]. Therefore, it is unclear whether the results can be generalized to individuals with MDD with elevated symptom intensity.

While we provide an analysis across multiple levels of behaviors by group, examining the influence of time-on-task and distractors, and conduct an exploratory correlational analysis, more in-depth integrative approaches combining these measures could further enhance our understanding of behavior in ADHD. For example, a machine learning approach similar to our analysis in Wiebe et al [18] could integrate the various parameters to classify between medicated versus unmedicated individuals with ADHD, and individuals with ADHD versus HC if combined with the dataset reported in Pakos et al [24]. Additionally, a more thorough analysis of distractor-related movement and gaze behavior, and their interplay during task errors, could illuminate potential underlying causes of said errors.

Moreover, while assessing the VEST’s discriminant validity in differentiating between ADHD and MDD represents an important step toward evaluating its potential use in a clinical context, future research might also explore the overlap and divergence of symptom profiles in individuals with comorbid ADHD and MDD. This could further inform the refinement and diagnostic use of VR-based assessments like the VEST. Consequently, although the actual clinical applicability of the VEST still requires further investigation, it is conceivable that it could be used, for example, as a complementary tool in differential diagnostics or for monitoring symptom severity or symptom changes associated with medication. Its administration is straightforward, and even if the space or financial resources for a fully equipped VR laboratory are not available, it would be possible to use the application on a smaller scale, for example, on a smartphone with an inexpensive VR headset alternative.

### Conclusion

This study examined, for the first time, the potential of our recently developed multimodal VEST to distinguish individuals with ADHD and MDD, and to detect medication-related effects in ADHD. An increase in PTV over time was observed in unmedicated ADHD and MDD participants, but not in those with medicated ADHD, suggesting a stabilizing effect of medication on attentional capacities. Moreover, medicated individuals with ADHD showed an increase in head rotation during distractions, and, on a trend level, both medicated and unmedicated individuals with ADHD showed increased distractor-associated head movements, an effect not observed in the MDD group. This finding underscores the potential of actigraphy in combination with distracting events during task performance as a tool for differentiating ADHD from other clinical populations. Additionally, exploratory correlational analyses showed correlations between various subjective and objective, as well as psychophysiological markers. While we also observed higher scores in task-related inattention, impulsivity, and emotional dysregulation in at least 1 ADHD group compared to MDD, no differences were found in gaze behavior and brain activity measured by fNIRS. To solidify the results and be able to reliably detect potential small size differences between the groups, and verify the task’s potential as an assessment tool with potential applications in research and a clinical context, higher-powered studies are needed.

## Appendix

Multimedia Appendix 1 Further specifications of hardware and software.

Multimedia Appendix 2 Missing data of each modality.

Multimedia Appendix 3 Tabular summaries of ANOVA key results.

Multimedia Appendix 4 Correlational analysis.

## Abbreviations

ADHD: attention-deficit/hyperactivity disorder
ADHS-SB: ADHD Self-Rating Scale
CPT: continuous performance task
dlPFC: dorsolateral prefrontal cortex
DP: distractor phase
DSM-5: Diagnostic and Statistical Manual of Mental Disorders (Fifth Edition)
fNIRS: functional near-infrared spectroscopy
HC: healthy controls
HMD: head-mounted display
ICD-10: International Statistical Classification of Diseases, 10th Revision
MDD: major depressive disorder
MDiff: mean difference
Mini-DIPS: Diagnostic Brief Interview for Mental Disorders
NDP: nondistractor phase
Oxy-Hb: oxyhemoglobin
PHQ-9: Patient Health Questionnaire-9
PTV: processing time variability
VEST: virtual email sorting task
VR: virtual reality
VRSQ: Virtual Reality Sickness Questionnaire
VSR: virtual seminar room

## Data Availability

The datasets generated or analyzed during this study are available from the corresponding author on reasonable request.
