# Supplementary material for: Multimodal Virtual Reality Assessment of Medication Effects in Attention-Deficit/Hyperactivity Disorder and Its Distinction From Depression: Cross-Sectional Study
Source: JMIR Hum Factors. 2026 Mar 2;13:e85351. doi: 10.2196/85351 (PMC12993270; doi:10.2196/85351)
Supplement: Multimedia Appendix 1 [file humanfactors_v13i1e85351_app1.docx]

**Supplementary Material 1: Further specifications of hardware and software**

**Apparatus and virtual environment**

We used the HTC VIVE Pro Eye head-mounted display (HTC Corporation, Taoyuan City, Taiwan), which features a 1440 × 1600 px image resolution per eye, a 110-degree field of view, and a nominal 90 Hz screen refresh rate.

The virtual office room was designed using Blender (The Blender Foundation, 2021). Additional textures and materials were sourced from public domain resources and ambientCG (Demes, 2021). The environment surrounding the office, visible through the windows, was created based on a CGTrader template. The co-workers, interior elements, and animations were assembled from a combination of commercial and non-commercial repositories, including Mixamo and CGTrader, or were self-created.

**Eye Tracking**

The built-in eye tracker of the HTC Vive Pro Eye is infrared-based and has a trackable field of view of 110° and an estimated accuracy of 0.5 – 1.1°. The position of both eyes were recorded and head movement was not restricted. Environmental lighting in VR was designed based on natural daylight and was kept constant. Participants were instructed to refrain from wearing eye makeup to optimize eye tracking performance and were asked to wear glasses instead of contact lenses if they had visual impairments. The online eye tracking data was collected from Unity via the SRanipal SDK version 1.3.1.1 (HTC Corporation, Taoyuan, Taiwan) and Tobii XR SDK 1.8.0 (Tobii Technology, n.d.) with a sampling rate of 50 Hz and streamed to LSL.

To access the participant’s raw eye tracking data, we used SRanipal SDK. We used the Tobii XR SDK’s IGazeFocusable interface to track the participant’s duration of momentary gaze focus on the canvas on which the task was displayed on (on task-gaze) and the duration spent looking away from the canvas (off-task gaze).

Offline analyses of the eye tracking data were performed in Matlab 2021b (The MathWorks Inc., Natick, MA, USA).

**fNIRS recording and analyses**

The participants' hemodynamic activity was recorded with a ~20.3 Hz sampling rate via twelve dual-tip LED optodes, consisting of 8 detectors and 4 sources, with wavelengths of 760 nm and 850 nm. We opted for a setup of a total of 10 active channels, with a source-detector distance of approximately 30 mm. To this end, we placed the sources at the 10-20 positions of the EEG cap at Fc1, Fc2, Cp1, and Cp2, and detectors at Fc3, Fc4, C1, C2, Cp3, Cp4, P1, and P2.

We used Matlab 2021b for the subsequent offline analyses. In the first step, using the toolbox QT-NIRS (Montero-Hernandez & Pollonini [48], 2020) the signal quality of the fNIRS channels of the left and right dlPFC, 1 and 6 was examined, with a quality threshold of 0.75 and a 5 s time window. For further fNIRS analyses, datasets with a signal quality that fell below threshold in one or both relevant channels were excluded. In a next step, further preprocessing was conducted via the Homer3 toolbox (Huppert et al [47], 2009): fNIRS raw data were first transformed to changes in optical density and motion artifacts were corrected via Wavelet analysis. Subsequently, a bandpass filter ranging from 0.01 to 0.5 Hz was applied to the data, after which changes in optical density were converted to hemoglobin concentration changes using the modified Beer–Lambert law (with a partial path length factor of 1).
