# Supplementary material for: Multimodal Virtual Reality Assessment of Medication Effects in Attention-Deficit/Hyperactivity Disorder and Its Distinction From Depression: Cross-Sectional Study
Source: JMIR Hum Factors. 2026 Mar 2;13:e85351. doi: 10.2196/85351 (PMC12993270; doi:10.2196/85351)
Supplement: Multimedia Appendix 2 [file humanfactors_v13i1e85351_app2.docx]

## Supplementary Material 2: Missing Data Of Each Modality

Absolute and relative amount of missing data of each modality investigated

| Measure | Missing data |
| --- | --- |
| Mean Error Rate | 62/62 (0%) |
| Mean Processing Time | 62/62 (0%) |
| Mean Processing Time Variability | 62/62 (0%) |
| Off-Task Gaze | 61/62 (1.6%) |
| Head Actigraphy | 62/62 (0%) |
| Task-related Impulsivity | 61/62 (1.6%) |
| Task-related Hyperactivity | 61/62 (1.6%) |
| Task-related Inattention | 61/62 (1.6%) |
| Task-related Emotional Dysregulation | 61/62 (1.6%) |
| Torso Actigraphy | 45/62 (27.4%) |
| Arm Actigraphy | 58/62 (6.5%) |
| fNIRS HbO1_1 | 29/46* (37.0%) |
| fNIRS HbO3_5 | 23/46* (50.0%) |

^*^Differences in fNIRS-measured brain activity was only computed for the two ADHD groups.

To examine whether missingness was systematically associated with group, gender, and age, we fitted binary logistic regression models predicting a missingness indicator (0=non-missing, 1=missing) for each variable [49]. The overall models were not significant for torso actigraphy (LR χ²(3)=1.188, *P*=.76) arm actigraphy (LR χ²(3)=4.490, *P*=.21), or Hb03_5 (LR χ²(3)=3.088, *P*=.38). In contrast, the model for Hb01_1 was significant (LR χ²(3)=10.902, *P*=.01), with gender emerging as a significant predictor (*P*=.02).

49 Heymans, M. W., & Twisk, J. W. (2022). Handling missing data in clinical research. *Journal of clinical epidemiology*, *151*, 185-188.
