# Supplementary material for: Multimodal Virtual Reality Assessment of Medication Effects in Attention-Deficit/Hyperactivity Disorder and Its Distinction From Depression: Cross-Sectional Study
Source: JMIR Hum Factors. 2026 Mar 2;13:e85351. doi: 10.2196/85351 (PMC12993270; doi:10.2196/85351)
Supplement: Multimedia Appendix 3 [file humanfactors_v13i1e85351_app3.docx]

**Supplementary Material 3: Tabular summaries of ANOVA key results**

ANOVA key results for VEST performance data

| **Condition** | **F** | **P** (Bonferroni corrected) | **η^2^_p_** |
| --- | --- | --- | --- |
| **Mean processing time** | | | |
| Block | 68.22 | <.001 | .54 |
| Phase | 23.99 | <.001 | .29 |
| Group | 1.49 | .24 | .05 |
| Block x Group | 0.43 | .65 | .01 |
| Phase x Group | 1.14 | .33 | .04 |
| Block x Phase | 4.00 | .05 | .06 |
| Block x Phase x Group | 0.06 | .94 | .00 |
| **PTV** | | | |
| Block | 8.39 | .005 | .12 |
| Phase | 0.46 | .50 | .01 |
| Group | 1.18 | .32 | .04 |
| Block x Group | 3.35 | .04 | .10 |
| Phase x Group | 5.09 | .009 | .15 |
| Block x Phase | 3.79 | .06 | .06 |
| Block x Phase x Group | 0.78 | .46 | .03 |
| **Error Rate (square-root transformed)** | | | |
| Block | 1.79 | .19 | .03 |
| Phase | 41.12 | <.001 | .41 |
| Group | .04 | .96 | .00 |
| Block x Group | 0.60 | .55 | .02 |
| Phase x Group | 1.69 | .19 | .05 |
| Block x Phase | 0.62 | .44 | .01 |
| Block x Phase x Group | 0.61 | .55 | .02 |

ANOVA key results for movement tracking data

| **Condition** | **F** | **P** (Bonferroni corrected) | **η^2^_p_** |
| --- | --- | --- | --- |
| **Head movement** | | | |
| Block | 60.12 | <.001 | .51 |
| Phase | 23.99 | .005 | .13 |
| Group | 0.48 | .62 | .02 |
| Block x Group | 0.86 | .43 | .03 |
| Phase x Group | 2.56 | .09 | .08 |
| Block x Phase | 1.38 | .24 | .02 |
| Block x Phase x Group | 0.55 | .58 | .02 |
| **Head rotation** | | | |
| Block | 17.33 | <.001 | .23 |
| Phase | 0.20 | .66 | .00 |
| Group | 3.08 | .053 | .10 |
| Block x Group | 1.34 | .27 | .04 |
| Phase x Group | 8.81 | <.001 | .23 |
| Block x Phase | 0.34 | .56 | .01 |
| Block x Phase x Group | 1.45 | .24 | .05 |
| **Arm movement (square-root transformed)** | | | |
| Block | 38.93 | <.001 | .41 |
| Phase | 0.02 | .90 | .00 |
| Group | 0.53 | .59 | .02 |
| Block x Group | 1.20 | .31 | .04 |
| Phase x Group | 0.26 | .77 | .01 |
| Block x Phase | 2.80 | .10 | .05 |
| Block x Phase x Group | 0.37 | .69 | .01 |
| **Arm rotation (square-root transformed)** | | | |
| Block | 13.29 | <.001 | .20 |
| Phase | 0.31 | .58 | .01 |
| Group | 1.73 | .19 | .06 |
| Block x Group | 1.29 | .28 | .05 |
| Phase x Group | 1.70 | .19 | .06 |
| Block x Phase | 5.41 | .02 | .09 |
| Block x Phase x Group | 0.27 | .76 | .01 |
| **Torso movement (square-root transformed** | | | |
| Block | 14.73 | <.001 | .26 |
| Phase | 0.01 | .91 | .00 |
| Group | 0.32 | .73 | .02 |
| Block x Group | 0.38 | .68 | .02 |
| Phase x Group | 0.74 | .49 | .03 |
| Block x Phase | 0.90 | .35 | .02 |
| Block x Phase x Group | 0.26 | .77 | .01 |
| **Torso rotation (square-root transformed)** | | | |
| Block | 0.56 | .46 | .01 |
| Phase | 4.30 | .04 | .09 |
| Group | 0.68 | .51 | .03 |
| Block x Group | 0.17 | .84 | .01 |
| Phase x Group | 0.15 | .86 | .01 |
| Block x Phase | 0.18 | .68 | .00 |
| Block x Phase x Group | 1.50 | .26 | .07 |

ANOVA key results for off-task gaze (square-root transformed)

| **Condition** | **F** | P (Bonferroni corrected) | **η^2^_p_** |
| --- | --- | --- | --- |
| Block | 0.50 | .48 | .01 |
| Phase | 4.72 | .03 | .08 |
| Group | 1.16 | .32 | .04 |
| Block x Group | 0.57 | .57 | .02 |
| Phase x Group | 0.46 | .64 | .02 |
| Block x Phase | 0.50 | .48 | .01 |
| Block x Phase x Group | 0.57 | .57 | .02 |

ANOVA key results for experience sampling analysis

| **Condition** | **F** | **P** (Bonferroni corrected) | **η^2^_p_** |
| --- | --- | --- | --- |
| **Inattention (square-root transformed)** | | | |
| Block | 9.52 | .003 | .14 |
| Group | 9.28 | <.001 | .24 |
| Block x Group | 0.07 | .93 | .00 |
| **Impulsivity** | | | |
| Block | 0.38 | .54 | .01 |
| Group | 6.16 | .004 | .18 |
| Block x Group | 1.83 | .17 | .06 |
| **Hyperactivity** | | | |
| Block | 16.87 | <.001 | .22 |
| Group | 0.54 | .59 | .02 |
| Block x Group | 2.53 | .09 | .08 |
| **Emotional dysregulation** |  |  |  |
| Block | 9.20 | .004 | .14 |
| Group | 3.74 | .03 | .11 |
| Block x Group | 1.67 | .20 | .05 |
