# Supplementary material for: Multimodal Virtual Reality Assessment of Medication Effects in Attention-Deficit/Hyperactivity Disorder and Its Distinction From Depression: Cross-Sectional Study
Source: JMIR Hum Factors. 2026 Mar 2;13:e85351. doi: 10.2196/85351 (PMC12993270; doi:10.2196/85351)
Supplement: Multimedia Appendix 4 [file humanfactors_v13i1e85351_app4.docx]

## Supplementary Material 4: Correlational Analysis

*Descriptive Statistics and Correlations for Study Variables*

| Variable | 1 | 2 | 3 | 4 | 5 | 6 | 7 | 8 | 9 | 10 | 11 | 12 | 13 | 14 |
| --- | --- | --- | --- | --- | --- | --- | --- | --- | --- | --- | --- | --- | --- | --- |
| 1. Mean PTV | — |  |  |  |  |  |  |  |  |  |  |  |  |  |
| 2. Mean Error | .079  (62) | — |  |  |  |  |  |  |  |  |  |  |  |  |
| 3. Mean PT | .174  (62) | .069  (62) | — |  |  |  |  |  |  |  |  |  |  |  |
| 4. Off-Task Gaze | .325*  (61) | .251  (61) | .056  (61) | — |  |  |  |  |  |  |  |  |  |  |
| 5. Head Movement | .348**  (62) | .08  (62) | .236  (62) | .423***  (61) | — |  |  |  |  |  |  |  |  |  |
| 6. Head Rotation | .262*  (62) | .038  (62) | .297*  (62) | .069  (61) | .505***  (62) | — |  |  |  |  |  |  |  |  |
| 7. Arm Rotation | .128  (58) | -.079  (58) | .128  (58) | .174  (57) | .291*  (58) | -.017  (58) | — |  |  |  |  |  |  |  |
| 8. Arm Movement | .154  (58) | -.062  (58) | -.001  (58) | .401**  (57) | .304*  (58) | .023  (58) | .403**  (58) | — |  |  |  |  |  |  |
| 9. Torso Movement | -.004  (45) | -.042  (45) | -.016  (45) | -.012  (44) | .167  (45) | .03  (45) | .184  (42) | .005  (42) | — |  |  |  |  |  |
| 10. Torso Rotation | -.155  (45) | .194  (45) | -.121  (45) | .016  (44) | .108  (45) | .111  (45) | -.03  (42) | -.142  (42) | .193  (45) | — |  |  |  |  |
| 11. Impulsivity | .115  (61) | .346**  (61) | -.066  (61) | .181  (61) | -.006  (61) | .004  (61) | .019  (57) | -.206  (57) | -.126  (44) | .109  (61) | — |  |  |  |
| Variable | 1 | 2 | 3 | 4 | 5 | 6 | 7 | 8 | 9 | 10 | 11 | 12 | 13 | 14 |
| 12. Hyperactivity | .202  (61) | .339**  (61) | .325*  (61) | .071  (61) | .345**  (61) | .298*  (61) | .182  (57) | .088  (57) | .058  (44) | .189  (61) | .224  (61) | — |  |  |
| 13. Inattention | .176  (61) | .186  (61) | .006  (61) | .280*  (61) | .161  (61) | -.079  (61) | .169  (57) | .18  (57) | -.11  (44) | -.001  (61) | .500***  (61) | .377**  (61) | — |  |
| 14. Emotional Dysregulation | -.03  (61) | .147  (61) | -.215  (61) | .294*  (61) | .047  (61) | -.047  (61) | -.064  (57) | -.02  (57) | -.035  (44) | .058  (61) | .395**  (61) | -.061  (61) | .308* | — |

*Note.*P<.05, **P<.01, ***P<.001*
